# Avian influenza virus H5N1 induces rapid interferon-beta production but shows more potent inhibition to retinoic acid-inducible gene I expression than H1N1 in vitro

**DOI:** 10.1186/1743-422X-9-145

**Published:** 2012-08-03

**Authors:** Zhiqiang Mi, Yonghong Ma, Yigang Tong

**Affiliations:** 1 Beijing Institute of Microbiology and Epidemiology, 20 Dong-Da Street, Beijing, Fengtai District, 100071, China; 2 Center for Disease Control and Prevention in Xinjiang Military Command, Xinjiang Uygur Autonomous Region, Xinjiang Uygur, 830000, China

**Keywords:** Avian influenza virus H5N1, Interferon-beta, RIG-I

## Abstract

**Background:**

The mechanisms through which the avian influenza virus H5N1 modulate the host’s innate immune defense during invasion, remains incompletely understood. RIG-I as a pattern recognition receptor plays an important role in mediating innate immune response induced by influenza virus. So, modulating RIG-I might be adopted as a strategy by influenza virus to antagonize the host’s innate immune defense.

**Methods:**

Here we chose an avian influenza virus A/tree sparrow/Henan/1/04 (H5N1) directly isolated from a free-living tree sparrow in Mainland China which is amplified in egg allantoic cavity, and researched its interferon induction and manipulation of RIG-I expression compared with influenza virus A/WSN/1933(H1N1), a well characterized mouse adapted strain, in human lung epithelial A549 cells and human embryonic kidney 293T cells.

**Results:**

Although the avian influenza virus H5N1 infection initiated a rapid IFN-beta production early on, it eventually presented a more potent inhibition to IFN-beta production than H1N1. Correspondingly, the H5N1 infection induced low level expression of endogenous RIG-I, an Interferon Stimulating Gene (ISG), and showed more potent inhibition to the expression of endogenous RIG-I triggered by exogenous interferon than H1N1.

**Conclusions:**

Manipulating endogenous RIG-I expression might constitute one of the mechanisms through which avian influenza virus H5N1 control the host’s innate immune response during infection.

## Background

Avian H5N1 influenza viruses were first recognized to be capable of causing human respiratory infection and disease in Hong Kong in 1997 when 18 documented human H5N1 infections with 6 fatalities were identified in conjunction with outbreaks of H5N1 disease among domestic poultry [1-3]. As a result of avian-to-human transmission, 373 laboratory-confirmed human cases (236 deaths) of H5N1 virus infection in 14 countries have been reported to the World Health Organization from 2003 to 18 March 2008 [4], highlighting the pandemic potential of H5N1 viruses and their growing influence on global public health. Since the 1997 H5N1 avian influenza virus transmission from chicken to human in Hong Kong, cross-species transmissibility of avian influenza virus H5N1 is a major concern in influenza research, but the underlying mechanism(s) of cross-species transmission and the heightened virulence of H5N1 viruses to humans remain largely unknown.

Since first identified in choriontic membranes of embryonated chicken eggs, interferon has been regarded as an important defence line in controlling virus infection [5-7]. In order to successfully invade and amplify, influenza virus has evolved to obtain so many strategies to fight against the above defence system during interplaying with hosts [8]. Retinoic acid-inducible gene-I(RIG-I) as an Pattern Recognition Receptor(PRR) sensing viral cytoplasmic RNA ligand located on the extreme upstream of signalling pathway mediating interferon production [9]. So RIG-I would be controlled or modified to downregulate interferon activation by some viruses such as influenza virus and Hepatitis C Virus [10], but the mechanisms have not been understood completely.

Considering the important role of innate immune response in controlling virus transmission and pathogenesis [11,12], in the current study, a novel genotype H5N1 strain A/tree sparrow/Henan/1/04(H5N1) isolated from a tree sparrow in Mainland China was chosen [13] and its interferon-beta (IFN-β) induction and modulation of endogenous RIG-I expression were compared with human influenza virus H1N1 in human lung epithelial cells. Although the avian influenza virus H5N1 infection caused rapid IFN-β production in early infection, in the late stage of infection, the H5N1 strain showed more potent IFN-β inhibition and induced low level endogenous RIG-I expression than human influenza virus H1N1. Our results further approved that through manipulating innate immune response even the expression of RIG-I, avian influenza virus H5N1 struggle to control the host’s innate immune defense system for facilitating its invasion.

## Results

### Avian influenza virus A/tree sparrow/Henan/1/04 (H5N1) induces more rapid IFN-β production than human influenza virus H1N1 in early infection

**Figure 1:**
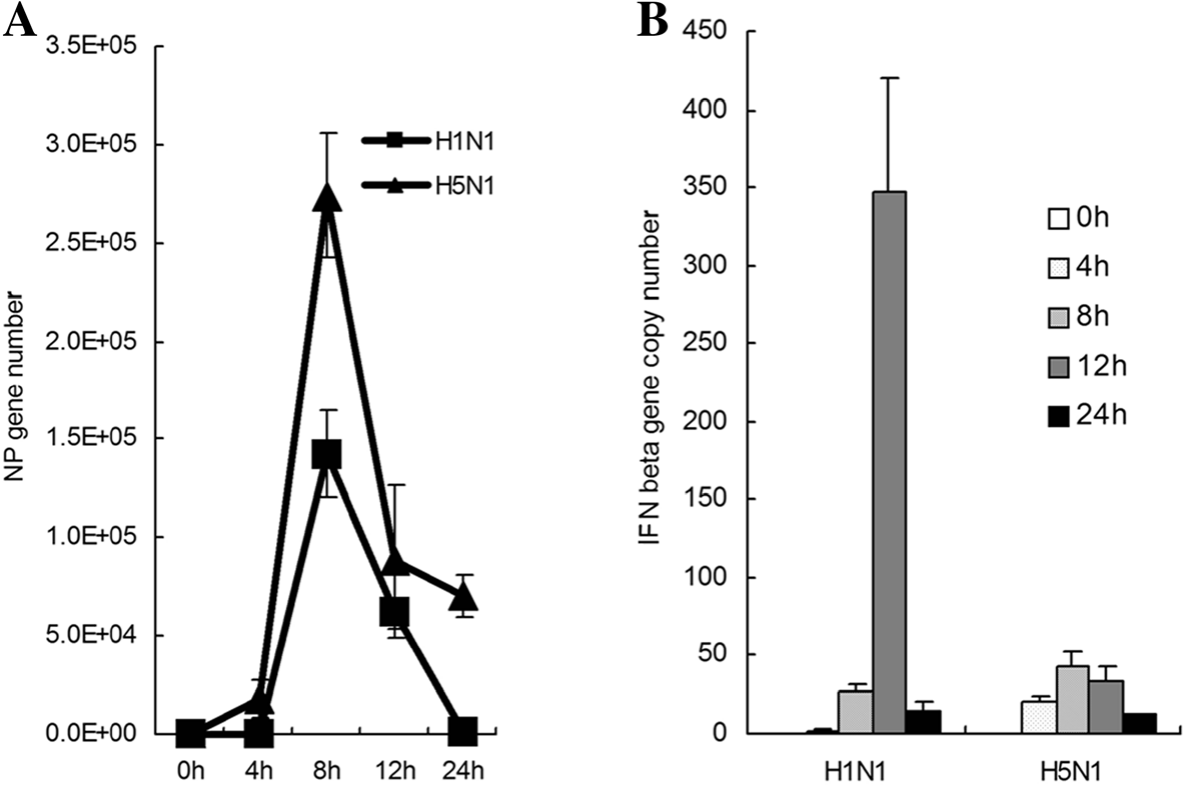
**Infection of avian influenza virus H5N1 resulted in robust NP gene expression and rapid IFN-β production.** A549 cells were infected with influenza viruses(m.o.i = 2). At the indicated time point, cells were collected and performed to extract total RNA. Quantitative RT-PCR was used to detect the copy numbers of the influenza virus *NP* gene, human *IFN-β* gene in A549 cells, and normalized with human β-actin gene copy numbers. Data are shown as mean ± SD of three independent experiments. ( **A**) The expression pattern of *NP* gene in A549 cells. ( **B**) The induction of *IFN-β* gene in A549 cells. Significance was determined using two-tailed Student’s *t* test (*, P < 0.05).

Innate immune response constitutes the first line of defense when a host encounters viral infections, but in the case of influenza virus infection, only low level or moderate IFN production is induced, and a different induction ability is apparent [14]. Our results also demonstrated this different inducing pattern. When normalized with multiplicity of infection, based on the level of nucleotide protein (NP) gene mRNA, we observed that the avian influenza virus A/tree sparrow/Henan/1/04(H5N1) infection was followed with more rapid and potent NP gene expression compared with human influenza virus H1N1 in the same multiplicity of infection (Figure 1A). In accordance with its robust gene expression in early infection (0–8 h), the avian influenza virus H5N1 induced rapid and potent IFN-β production at 4h post infection compared with H1N1, but this pattern abruptly went down in late infection (12–24 h) (Figure 1B).

### Avian influenza virus H5N1 infection induced low level endogenous RIG-I expression

**Figure 2:**
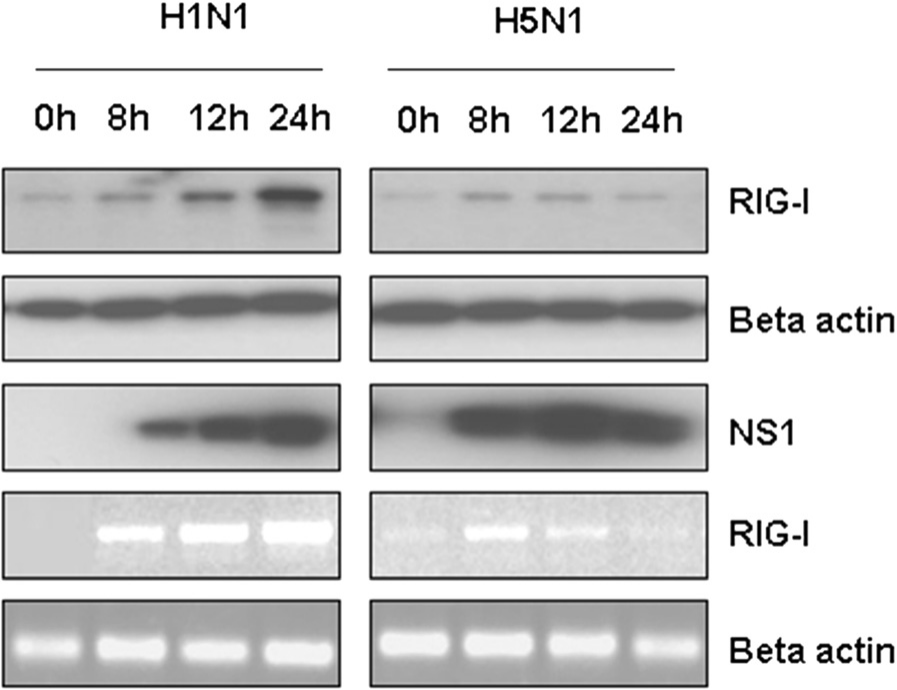
**H5N1 infection induced low level endogenous RIG-I expression.** The A549 cells were infected with H5N1 or H1N1 (m.o.i = 2) for 1 h, then the supernatant was replaced with fresh medium and culturing was continued. At the indicated time, the cells were collected. Half of the cells were used to detect endogenous RIG-I expression, and human β-actin was used as the loading control. The other half of the cells was used to extract total RNA and synthesize cDNA as previously demonstrated. Semiquantitative PCR was used to detect the RIG-I and human β-actin mRNA level.

RIG-I not only functions as a PRR to initiate interferon production but also inducible by endogenous or exogenous interferon as an Interferon Stimulated Gene (ISG) [15,16]. Previous studies have demonstrated that although single strand RNA (ssRNA) from influenza virus can be recognized by RIG-I [17] and activate downstream signaling molecules, influenza virus infection only induces weak IFN production because NS1 protein blocks the IFN signaling pathway [18]. Here we observed that although A/tree sparrow/Henan/1/04(H5N1) effectively infected human lung epithelial cells and induced rapid IFN-β production during early infection (0-4h) (Figure 1B), its infection only induces low level endogenous RIG-I expression compared with H1N1 in A549 cells based on western blot and RT-PCR and that they are in agreement (Figure 2).

### RIG-I is involved in recognizing H5N1 to produce interferon-beta

**Figure 3:**
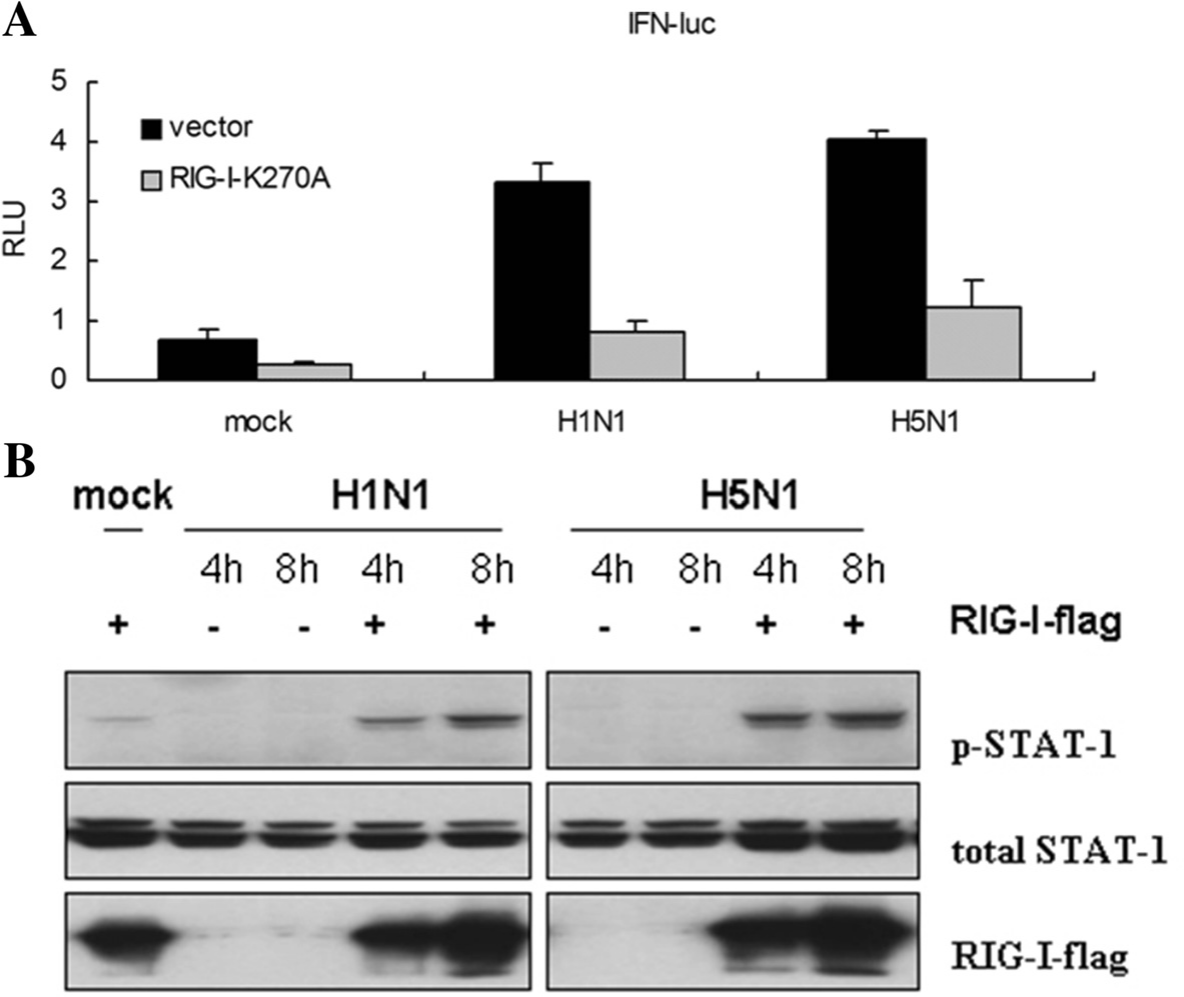
**Involvement of RIG-I in recognizing influenza virus.** ( **A**) The A549 cells were cotransfected with the *IFN-luc* reporter gene, pCMV-renilla, and RIG-I-flag K270A. At 16 h post transfection, the cells were infected with the H5N1 or H1N1 (m.o.i = 2). At 12 h post infection, the cells were collected and luciferase reporter activity was measured with the Dual-Luciferase Reporter Assay System. In all cases, the data are shown as mean ± SD of triplicate samples of a representative from three independent experiments. ( **B**) Overexpressing RIG-I increases STAT-1 phosphorylation induced by H1N1 or H5N1 infection. HEK293T cells were transfected with RIG-I flag. At 16 h posttransfection, cells were infected with indicated influenza virus (m.o.i = 2). Then the cells were collected at 4 h and 8 h post infection, and western blot was used to detect STAT-1 phosphorylation, total STAT-1, RIG-I, and NS1 expression.

Innate immune pattern recognition receptors exist at the extreme upstream of IFN signaling pathway, and different PRRs recognize the specific components from invading microbial pathogens and initiate innate immune response [19-22]. RIG-I has been identified as the cytosolic innate immune PRR that mediates IFN production through recognizing 5-termial ppp single strand RNA [23]. Previous studies indicated that RIG-I was responsible for mediating IFN production when sensing influenza virus in epithelial cells [24]. Pretreating the A549 cells with IFN-α or TNF-α increases RIG-I expression and enhances the IFN production induced by influenza virus infection [15,16]. Our results also proved that as with other influenza virus strains, RIG-I was involved in the recognition of A/tree sparrow/Henan/1/04(H5N1) infection. Over expressing RIG-I K270A (a dominant negative construct of RIG-I) significantly inhibits the IFN-β reporter response induced by A/tree sparrow/Henan/1/04(H5N1) or H1N1 infection (Figure 3A). Meanwhile, over expressing wild type RIG-I in 293T cells significantly increased STAT-1 phosphorylation in a time dependent manner with increasing phosphorylation seen for 8 hours versus 4 hours, which signals in IFN production (Figure 3B).

### The infection of H5N1 exerted potent blockage to endogenous RIG-I expression when treated with exogenous interferon

**Figure 4:**
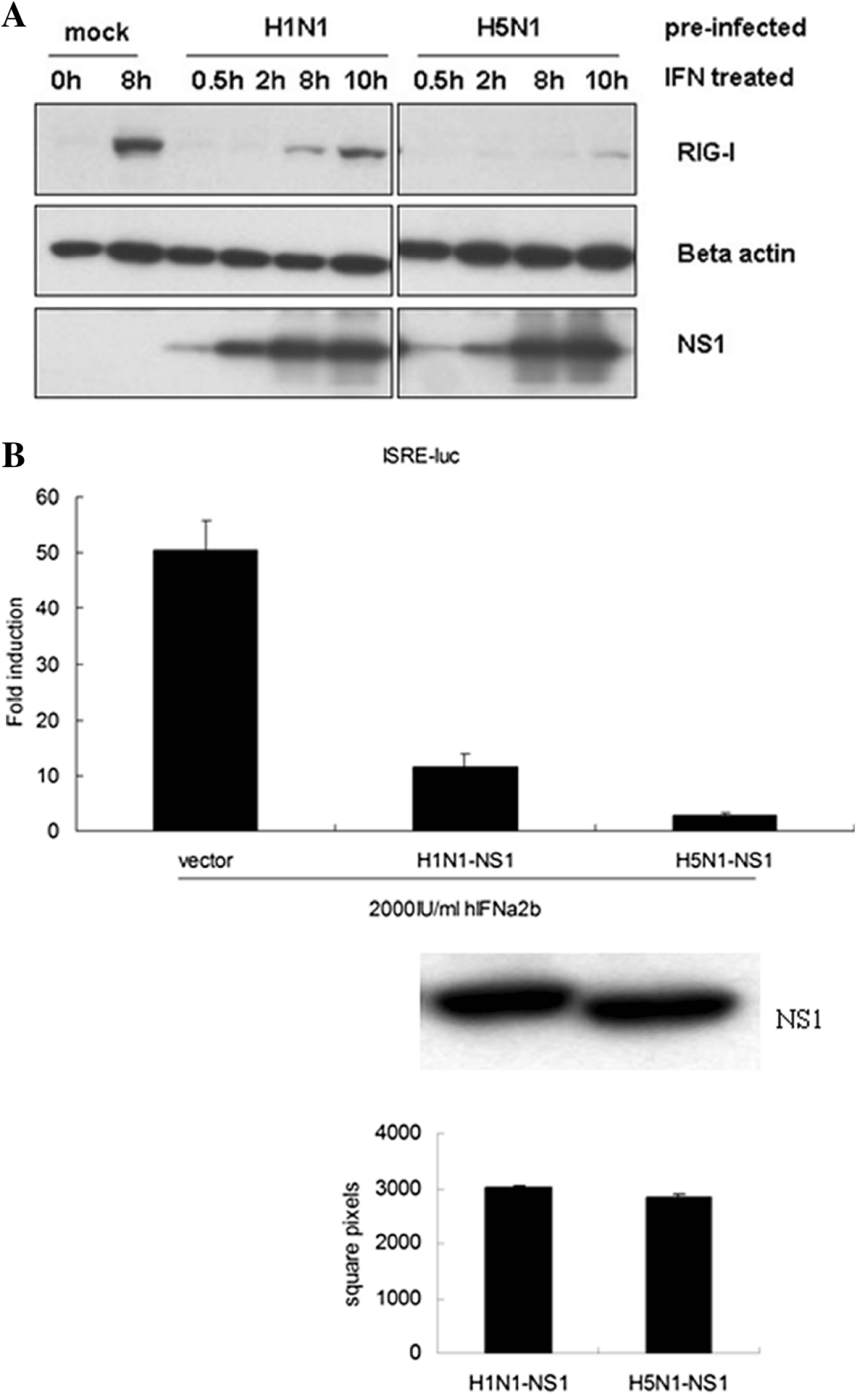
**H5N1 infection or NS1 overexpression showed potent inhibition to exogenous IFN.** ( **A**) HEK293T cells were pre-infected as indicated. At 4 h post infection, the cells were treated with 2000 IU/ml of hIFNα2b (InvivoGen, San Diego, CA) for the indicated time. The expression of endogenous RIG-I and NS1 were analyzed with western blot, beta-actin as loading control. ( **B**) HEK293T cells were co-transfected with the ISRE-luc reporter gene, pCMV-renilla, and NS1 constructs. At 16 h post transfection, cells were treated with 2000 IU/ml of hIFNa2b for 8 h. The cells were collected, and luciferase reporter activity was measured with the Dual-Luciferase Reporter Assay System. Fold induction is the ratio of the activity induced by the hIFNa2b treatment to that of the untreated control. In all cases, data are shown as mean ± SD of triplicate samples. The expression level of NS1 constructs in HEK293T was shown in western blot and measured with Scion image software (National Institute Health).

In order to further assess the ability of antagonizing interferon and manipulation to endogenous RIG-I expression, 293T cells were pre-infected with H5N1 and H1N1, and then treated with human IFN-α2b. The results indicated that although both of the influenza viruses showed somewhat inhibition to exogenous interferon and decreaed endogenous RIG-I expression, H5N1 gave more potent inhibition to endogenous RIG-I expression induced by exogenous interferon than H1N1 (Figure 4A). NS1 has been recognized as the key component acting as antagonizing interferon. In this assay, we also found that as shown in the case of infection, NS1 from H5N1 presented more potent inhibition to the response of IFN-stimulated response element (ISRE) reporter gene induced by exogenous interferon than H1N1 (Figure 4B).

## Discussion

The critical role of the IFN system in controlling influenza virus transmission was first demonstrated in a classical experiment wherein mice infected with influenza virus and injected with anti-IFN serum were observed to produce higher titers of the virus and an exacerbated pathology [25]. Subsequent studies in knockout mice with defects in their IFN system have confirmed this basic observation [26]. Recent studies in which the IFN-inducing capacity varied in genetically related A-type influenza virus showed that the strain capable of producing the most IFN was attenuated in its pathogenesis in mice [27,28]. The above researches indicate that IFN plays an important role in limiting human influenza virus or avian influenza virus pathogenesis or transmission.

Since the first reported human case of avian influenza virus H5N1 infection in Hong Kong in 1997 [2], the avian to human or human to human transmission of H5N1 poses a potential threat to human health. Aquatic birds are considered the natural host of avian influenza virus H5N1 [29]. But recently, some terrestrial birds, such as tree sparrows, have been found carrying H5N1 with no symptoms [30]. This indicates that the host range of avian influenza virus maybe expanding. In addition, some new H5N1 genotypes are emerging through gene reassortment [13,31]. How those avian influenza viruses pose a threat to humans deserves further study. In the current assay, we chose a new genotype avian influenza virus A/tree sparrow/Henan/1/04(H5N1) isolated from a free-living tree sparrow in Henan Province of Mainland China and studied its modulation of IFN-β and RIG-I in mammalian cells.

In contrast to human influenza virus H1N1, A/tree sparrow/Henan/1/04(H5N1) infection in mammalian cells resulted in rapid IFN-β production during the early stage of infection, but in the late stage, it significantly inhibited the amplification of IFN-β production and showed the same pattern as the Indian isolate of pandemic (H1N1) 2009 influenza virus [32]. Previous studies have demonstrated that highly pathogenic avian influenza virus H5N1 replicated more efficiently than human influenza viruses due to its PA, PB1, and PB2 [33-35]. Our results also proved that A/tree sparrow/Henan/1/04(H5N1) was consistent with other H5N1 strains and that it rapidly and efficiently amplified in mammalian cells, which resulted in the rapid activation of IFN-β signaling in early infection. However, accompanying rapid replication and viral cytoplasmic RNA accumulating, why the rapid IFN-β production was followed with sharp downregulation during H5N1 infection is unknown. In this study, we observed that although A/tree sparrow/Henan/1/04(H5N1) infection caused rapid IFN-β production and STAT-1 phosphorylation during the early stage of infection, it only induced low level endogenous RIG-I expression in comparison to H1N1 in A549 cells. Furthermore, when preinfecting the 293T cells, H5N1 significantly inhibited the endogenous RIG-I expression induced by exogenous interferon than H1N1. Previous investigation showed that low level RIG-I existed in quiescent epithelial cells and ready to sense invading RNA [16]. When the primary interferon produced, it increased RIG-I expression, which again participates in sensing invading RNA and amplifying interferon production. So inhibiting RIG-I expression unavoidly interrupt the above positive feed-back loop. According to these results we assume that manipulating RIG-I expression might be adopted by influenza virus at least as one of strategies antagonizing host’s innate immune response.

Although a latest literature found the polymerase of H5N1 was involved in antagonzing interferon production in chicken macrophage HD-11 cells [36], the NS1 has been regarded as the key component inhibiting interferon production through targeting many aspects of interferon signalling pathway [37]. In this study NS1 from H5N1 showed more potent inhibition to exogenous interferon than NS1 from H1N1 in the same level of expression. The same level of NS1 from different subtype or strain of influenza virus exhibiting diverse antagonizing capacity might attribute to its property [38,39]. So, the accumulating NS1 protein during influenza virus infection might be responsible for the differences in inducing interferon or RIG-I expression. Based on these results, during the early stage of infection, H5N1 replicates more efficiently than H1N1 and induced rapid IFN-β production, but in the late stage of infection, it significantly inhibits IFN-β production with NS1 accumulation as showed in influenza C virus NS1 [40].

## Conclusion

Previous studies showed that multistep inhibition contributes to the capacity of NS1 to antagonize the innate immune response of the host, such as through the inhibition to PKR signaling by binding to double strand RNA (dsRNA) [41,42], inhibition to nuclear export of polyadenylated host mRNA [43], and inhibition to mRNA splicing and downregulation of IFN production by interacting with CPSF (cleavage and polyadenylation specificity factor) [44-46]. Here we showed that inhibiting endogenous RIG-I expression is another mechanism through which A/tree sparrow/Henan/1/04 (H5N1) antagonizes IFN-β production. This further confirms that the influenza virus inhibits the host innate immune response through multiple steps and aspects.

## Methods

### Virus and cells

A/tree sparrow/Henan/1/04(H5N1) (kindly provided by Professor Li TX, State Key Laboratory of Virology, Wuhan Institute of Virology, Chinese Academy of Sciences) and A/WSN/1933(H1N1) purchased from the American Type Culture Collection (ATCC, Manassas, VA) were propagated in 10-day-old embryonated chicken eggs from specific-pathogen-free flocks (Beijing Merial). Each egg was injected with 0.1 ml of phosphate-buffered saline (PBS) containing ~10^3^ infectious particles, incubated at 35°C with forced air circulation and egg rotation, and maintained at 4°C for 12 h before harvesting. The allantoic fluid was harvested separately from each egg. Those with high hemagglutinin activity were pooled, and aliquots were prepared and stored at −70°C. The hemagglutinin titer of the above subtype of influenza virus was measured with 1% hamster blood cell in a U-shaped plate. Human lung carcinoma cell A549, human embryonic kidney 293T cell (HEK293T) and Madin-Darby Canine Kidney Cell (MDCK) cell were purchased from ATCC and maintained in minimal essential medium (MEM, HyClone). All cell cultures were supplemented with 10% fetal calf serum (PAA Laboratories, Germany), 100 U penicillin, and 100 μg streptomycin per milliliter (Sigma-Aldrich, St. Louis). The cells were incubated at 37°C and 5% carbon dioxide.

### Tansfection

Transient transfection of A549 cells with the indicated plasmids was conducted with Lipofectamin 2000 (Invitrogen) according to the manufacturer’s recommendation. Transient transfection of HEK293T cells with indicated plasmids was performed routinely with calcium phosphate method. In each experiment total amounts of DNA in each sample were kept constant by supplementation with the appropriate empty parental expression vector.

### Luciferase reporter assay

IFN-luc and ISRE-luc luciferase reporter plasmids were described previously [47]. Briefly, the reporter plasmids were co-transfected with the indicated constructs into A549 cells or HEK293T cells. For normalization of transfection efficiency, Renilla luciferase plasmid was co-transfected as internal control of transfection efficiency. Luciferase activities were measured and normalized as per instructions by the manufacturer (Dual-Luciferase reporter assay system, Promega, WI).

### Plaque assay

Six-well tissue culture plates were seeded at 1 × 10^6^ MDCK cells per well. At 100% confluence, the cells were washed twice with PBS. Serial dilutions of the virus (10^-2^ to 10^-7^) were prepared in MEM containing 20 μg/ml TPCK–trypsin (Sigma-Aldrich). After infection of the wells with 0.5 ml of each dilution, the plates were incubated at 37°C for 1.5 h. The wells were aspirated to remove residual viral solution. Each well was then immediately covered with 2.5 ml MEM containing 0.8% agar and 20 μg/ml TPCK–trypsin. About 5 min later, the agar solidified. The plates were placed in the incubator. After 24 hours, 1.5 ml of 1× neutral red was added per well and incubated for 2 h. Then, the neutral red was pipetted without touching the agar. The quantity of plaque was counted, and the titer of virus was calculated as plaque-forming unit (PFU) per milliliter.

### Virus infection

For influenza virus infection, 2 × 10^6^ A549 or 293T cells were seeded in 60 mm-diameter dish. At 24 h post seeding, cells were washed with PBS and infected with influenza viruses (m.o.i = 2) in complete culture medium for 1 h, then virus-containing solutions were removed and cells were continuously cultured in complete culture medium in 5% CO_2_ incubator at 37°C. For mock infection, the same amount of allantoic fluid from non-inoculated 10-day-old chicken embryos was used to infect the A549 cells.

### Quantitative real-time RT-PCR

The complementary DNA (cDNA) number of individual avian influenza virus genes when oligo(dT) is used for reverse transcription can be used to designate the viral infection and amplification in host cells. For cDNA synthesis, total RNA was extracted from the cells infected with the indicated influenza virus using Trizol (Invitrogen, Carlsbad, CA), and reverse transcription was performed with M-MLV reverse transcriptase and oligo(dT)15 (Promega). Single-color real-time PCR System (Bio-Rad, Hercules, CA) was used to detect the levels of influenza virus NP mRNA and IFN-β mRNA. The real-time PCR primers were designed as the following: *NP* gene, forward primer: 5′-GAC GAA AAG GCA ACG AAC C-3′, reverse primer: 5′-TCA TAC TCC TCT GCA TTG TCT C-3′; human IFN-β gene, forward primer: 5′-AAA CTC ATG AGC AGT CTG CA-3′, reverse primer: 5′-AGG AGA TCT TCA GTT TCG GAG G-3′; human β-actin gene, forward primer: 5′-AGC GAG CAT CCC CCA AAG TT-3′; reverse primer: 5′-AGG GCA CGA AGG CTC ATC ATT-3′.

### Western blot

The cells were lysed in ice-cold lysis buffer containing 50 mM Tris–HCl (pH 7.5), 150 mM NaCl, 1% Nonidet P-40, 0.5% sodium deoxycholate, 0.1% sodium dodecyl sulfate (SDS), and a protease inhibitor cocktail (Sigma-Aldrich). Protein concentrations were determined using the Bradford reagent protocol (Bio-Rad). Equal amounts of proteins were loaded on an SDS-polyacrylamide gel, separated with electrophoresis, and blotted with the indicated antibodies according to the manufacturer's instructions. Immunoreactive proteins were detected with monoclonal antibody against RIG-I (Alme-1; Alexis, Plymouth Meeting, PA), rabbit anti-STAT1, phospho-STAT-1 (Tyr701) (58D6; Cell Signaling Technologies, Beverly, MA), anti-flag M2 monoclonal antibodies (Sigma-Aldrich), anti-NS1 monoclonal antibodies (sc-130568; Santa Cruz Biotechnology), anti-human β-actin monoclonal antibodies (sc-130301; Santa Cruz Biotechnology), and followed by horseradish peroxidase-conjugated secondary antibodies (Cell Signaling Technologies).

## Competing interests

The authors declare that they have no competing interests.
